# Case report: *Bordetella holmesii*: A rare pathogen causing infective endocarditis associated glomerulonephritis

**DOI:** 10.3389/fped.2022.1093300

**Published:** 2023-01-16

**Authors:** Tara Gavcovich, Malek Al Barbandi, Pamela Millan, Elizabeth Isner, Marissa J. Defreitas, Wendy Glaberson, Chryso P. Katsoufis, Jayanthi Chandar, Vaka Sigurjonsdottir, Ivan A. Gonzalez, Sethuraman Swaminathan, Yiqin Zuo, Carolyn L. Abitbol, Wacharee Seeherunvong

**Affiliations:** ^1^ Department of Pediatrics, Holtz Children’s Hospital, Miami, FL, United States; ^2^ Division of Pediatric Nephrology, Department of Pediatrics, Leonard M. Miller School of Medicine, University of Miami, Miami, FL, United States; ^3^ Division of Pediatric Infectious Disease, Department of Pediatrics, Leonard M. Miller School of Medicine, University of Miami, Miami, FL, United States; ^4^ Division of Pediatric Cardiology, Department of Pediatrics, Leonard M. Miller School of Medicine, University of Miami, Miami, FL, United States; ^5^ Department of Pathology, Leonard M. Miller School of Medicine, University of Miami, Miami, FL, United States

**Keywords:** endocarditis (infectious), glomerulonephritis, acute kidney injury, *Bordetella holmesii*, hematuria

## Abstract

Infective endocarditis (IE) can cause multiorgan dysfunction and chronic kidney disease, in addition to cardiac sequelae. The presentation may be vague and can manifest as acute glomerulonephritis. While the most common pathogens of infective endocarditis are *Staphylococcus* and *Streptococcus* species, we report a rare pathogen *Bordetella holmesii* causing infective endocarditis associated glomerulonephritis. A 20-year-old male patient with tetralogy of Fallot with pulmonary atresia and aortopulmonary collaterals underwent several cardiac surgeries including prosthetic pulmonary valve replacement in the past. He was admitted for 3 days at an outside hospital for fever, cough, and hemoptysis, and diagnosed with streptococcal pharyngitis, for which he received antibiotics. Five weeks later, he presented to our institution with lower extremity edema and gross hematuria. On examination, he was afebrile, normotensive, had a 7-kg weight gain with anasarca, and a systolic murmur, without rash. Investigations revealed elevated serum creatinine, nephrotic range proteinuria, hematuria, and hypocomplementemia, consistent with acute glomerulonephritis. Given his cardiac history, blood cultures were collected from three sites. Broad-spectrum antibiotics were initiated when he subsequently developed fever. Renal pathology on biopsy showed diffuse proliferative immune complex-mediated glomerulonephritis. Transesophageal echocardiogram visualized a vegetation on the pulmonary valve. *Bordetella holmesii* was ultimately cultured from the prior and current hospitalization. A serum sample detecting microbial cell-free DNA sequencing confirmed *Bordetella holmesii* at very high levels. After completing 6 weeks of intravenous antibiotics with concurrent angiotensin receptor blockade, his kidney function recovered with improvement in hypocomplementemia and proteinuria. This case report highlights the early recognition and comprehensive evaluation of a rare organism causing IE-associated GN, which allowed for renal recovery and preserved cardiac function.

## Introduction

Early recognition of infective endocarditis (IE) is essential, as it can cause multiorgan failure, cardiac decompensation, and advanced kidney disease if not properly managed (1, 2). The presentation of IE ranges from vague symptoms including fever, fatigue, or weight loss to septic shock and occasionally with kidney manifestations (1, 3).

Manifestations of renal involvement can include hematuria, proteinuria, acute kidney injury, infarction from septic emboli, damage secondary to deposition of immune complexes, direct immune-mediated destruction, and secondary interstitial nephritis from antibiotic and drug toxicities (3, 4). The most common pathogens of IE are *Staphylococcus* and *Streptococcus* species (1, 3, 4).

*Bordetella holmesii* (*B. holmesii*) has been implicated in IE; however, the association with glomerulonephritis (GN) has not been previously reported. In this case, we describe the rare pathogen *B. holmesii* causing IE-associated GN in a 20-year-old male patient confirmed by kidney biopsy, echocardiogram, and blood culture.

## Case description

The patient is a 20-year-old Hispanic male, with a diagnosis of tetralogy of Fallot, pulmonary atresia, and multiple aortopulmonary collaterals status post corrective surgical repair. He has had four previous cardiac surgeries including a central aortopulomonic shunt and coil embolization of aortopulmonary collateral vessels in the neonatal period, followed by a complete repair of pulmonary atresia and septal defect at 10 months of age. At 3 years of age, he required a bioprosthetic pulmonary valve replacement. He subsequently developed significant obstruction across the bioprosthetic valve and underwent his last cardiac surgery at 9 years of age for another bioprosthetic pulmonary valve replacement. Since then, the patient had been healthy per NYHA functional class I.

Five weeks prior to presentation at our institution, he was hospitalized at an outside hospital for 3 days with fever, cough, and a single episode of hemoptysis. He was treated with intravenous ceftriaxone and oral doxycycline and was discharged to complete a 7-day course of clindamycin with a diagnosis of streptococcal infection. After disposition, the mother was notified of a positive blood culture, but the patient had been well without further fever or hemoptysis so it was thought to be a contaminant. The patient was subsequently seen by his primary cardiologist within a week of discharge for further evaluation. His physical examination and a transthoracic echocardiogram remained at his baseline without any vegetation.

He then presented to our institution 5 weeks after his initial episode of fever and cough with a 2-day history of lower extremity edema and gross hematuria. He reported no fever, rash, joint pain, or dysuria.

In addition to his complex cardiac disease and prior surgical histories as above, the patient has congenital hypothyroidism, learning disability, and required special education. He has no history of opportunistic infections. He was taking levothyroxine and no other medications.

On physical examination, the vital signs showed a temperature of 37.4°C, heart rate of 78 beats per minute, blood pressure of 114/59 mmHg, and respiratory rate of 20 breaths per minute with an oxygen saturation of 100%. His weight was 71.3 kg, as compared to 64 kg from 1 month prior. He was nontoxic appearing, had audible crackles in bilateral lung bases, and had a regular heart rate and rhythm with a 4/6 systolic ejection murmur without gallop. He had abdominal ascites and lower extremity edema. No petechial rash, splinter hemorrhage, or Roth spots were noted.

Further evaluation showed an enlarged cardiac silhouette with bilateral interstitial opacities on chest x-ray. Transthoracic echocardiogram revealed a dilated right atrium, decreased right ventricular systolic function, normal left ventricular dimensions with mildly depressed systolic function, and moderate pulmonary valve stenosis without pulmonary regurgitation, which was not significantly different from previous studies. Retroperitoneal ultrasound revealed enlarged and echogenic kidneys.

The initial laboratory investigations showed mildly elevated white blood cells of 11,100 cells/mm^3^, with neutrophils predominant (85%), normocytic anemia with hemoglobin of 7.8 g/dl, and normal platelet count of 168,000 cells/mm^3^. There was no evidence of hemolysis with normal reticulocyte count and bilirubin as well as an elevated haptoglobin from acute phase reaction. He had elevated blood urea nitrogen and creatinine to 57 and 3.4 mg/dl, respectively. He had hematuria and nephrotic range proteinuria, with urine protein to creatinine ratio of 1.9 mg/mg creatinine. He had hypocomplementemia with complement C3 of 52 mg/dl and C4 of 5 mg/dl (Table 1). He had negative anti-streptolysin, low titers of antinuclear antibody at 1:40, and anti-neutrophil cytoplasmic antibody (ANCA) at 1:20, with a positive ANCA-PR3. His anti-double-stranded DNA was negative. Viral screening was negative for HIV antibody, hepatitis B surface antigen, and hepatitis C antibody.

**Table 1:** Biochemistry progression.

	Normal	Initial Lab	Day 4	Week 1	Week 3	Week 6	Week 12
CRP (mg/dl)	<0.5	8.9	6.7	1.9	2.2	<0.5	—
S Cr (mg/dl)	0.6–1.2	3.4	3.3	2.3	1.7	1.0	1.0
C3 (mg/dl)	90–180	52	49	—	—	87	143
C4 (mg/dl)	10–40	5	3	—	—	15	27
Up/c (mg/mg)	<0.2	1.9	1.7	2.6	6.5	2.2	0.6
U RBC/HPF	0–3	>182	71	35	139	16	3–10

CRP, C-reactive protein; S Cr, serum creatinine; C3, complement C3; C4, complement C4; Up/c, urine protein to creatinine ratio; U RBC/HPF, urine red blood cell per high power field; mg/dl, milligram per deciliter; mg/mg, milligram per milligram.

Given early concern for IE, blood culture was initially obtained in the emergency room, and two subsequent samples were drawn when the patient developed fever after admission. Empiric intravenous broad-spectrum antibiotics with vancomycin and cefepime were initiated along with supportive therapy including diuretics.

Within 72 h of hospitalization, one of three blood culture samples isolated an organism, but it was initially reported as *Bacillus* species. Upon review of his previous records, the blood culture from the outside hospital isolated *B. holmesii*, identified by matrix-assisted laser desorption/ionization-time of flight (MALDI-TOF) mass spectrometry (MS). It was sensitive to cefepime, ceftazidime, ciprofloxacin, gentamycin, levofloxacin, meropenem, but resistant to aztreonam. Given the conflicting results and the rarity of the pathogen, another serum sample was sent for microbial cell-free DNA sequencing. This specialized test confirmed *B. holmesii* at 12,474 DNA molecules per microliters, the highest number of copies ever detected of this organism by the testing facility. Additionally, the isolation of the positive blood culture at our institution speciated and was finalized as *B. holmesii* as well, as opposed to the initial report of *Bacillus* species, leading to three confirmed positive blood cultures of the rare pathogen.

**Figure 1:**
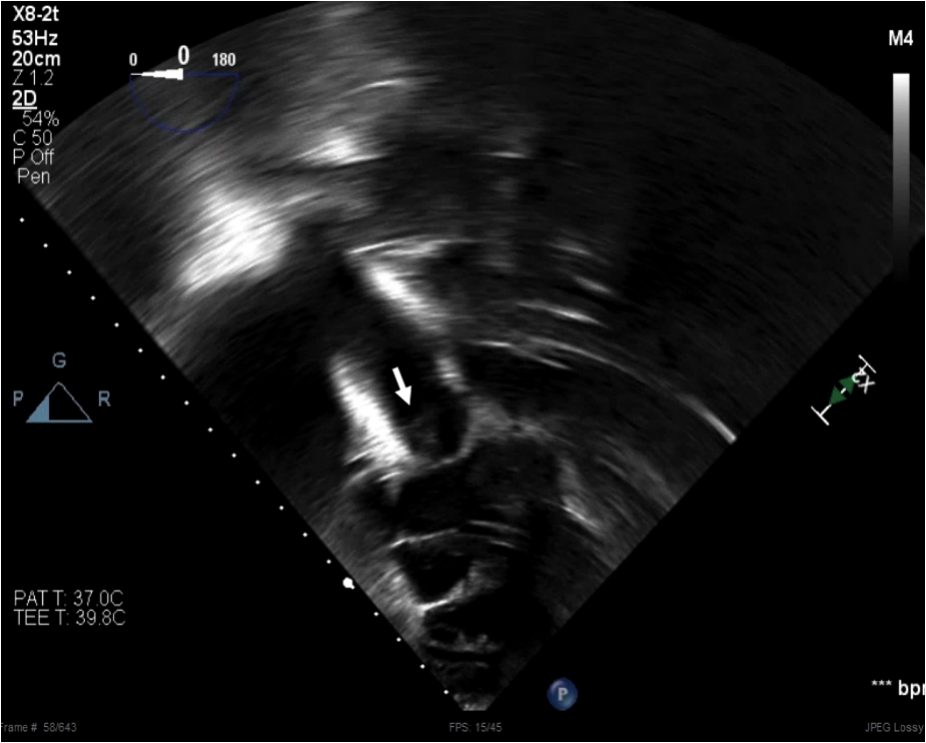
A mobile structure (arrow) visualized in the pulmonary valve from trans-gastric view on transesophageal echocardiogram. The structure moved with the valve, well attached to the leaflets.

To further confirm the diagnosis of IE, transesophageal echocardiogram revealed a mobile structure, well attached to and moved with the pulmonary valve leaflets, concerning for a pulmonic valve vegetation (Figure 1). Based on his echocardiogram findings, microbiology results, clinical presentation, and medical history, he met the definitive diagnosis for infectious endocarditis by the Duke criteria (5, 6).

**Figure 2:**
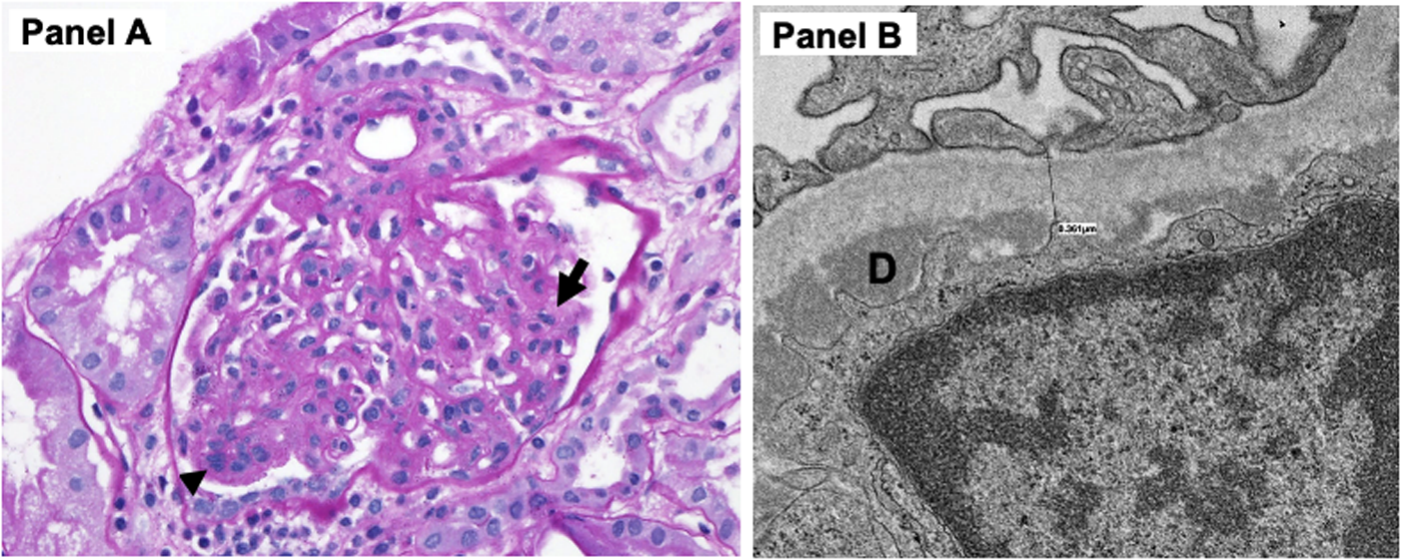
(**A**) Light microscopy demonstrated increased mesangial cellular and matrix expansion (arrow) as well as endocapillary hypercellularity (arrowhead) with scattered neutrophils within capillary lumens. (**B**) Electron microscopy revealed focal foot process effacement, subendothelial deposits (D). There were also multiple mesangial deposits (not shown) but no subepithelial deposits noted.

Kidney biopsy was obtained on the fourth day of admission, the same time as the transesophageal echocardiogram. The pathology demonstrated 14 glomeruli by a light microscope with diffuse increase in mesangial cellularity. Three glomeruli showed segmental endocapillary hypercellularity with scattered neutrophils within capillary lumens, and one additional glomerulus appeared to contain a cellular crescent. By immunofluorescence, there were IgM and C3 codominant (3+) near-global granular mesangial staining with segmental capillary loop extension with slightly weaker IgG and C1q (2+ to 3+). Electron microscopic examination revealed frequent mesangial/paramesangial deposits and scattered subendothelial deposits with no subepithelial deposits. The pathological diagnosis was diffuse proliferative immune complex-mediated glomerulonephritis (Figure 2).

The patient became afebrile within 1 day of empiric antibiotics, with resolution of edema and improvement of kidney function during the 12-day hospitalization. All subsequent blood cultures continued to be sterile. The patient completed a 6-week course of targeted antibiotics (cefepime) with concurrent with angiotensin receptor blockade for proteinuria. Subsequent echocardiogram 5 weeks after the onset of IE revealed a stable cardiac condition at his baseline with moderate pulmonary valve stenosis and no vegetation seen. The progressive improvement of inflammatory markers, kidney function, hypocomplementemia, and proteinuria during treatment and follow-up are shown in Table 1.

## Discussion

We describe, for the first time, a case of *Bordetella holmesii* causing IE-associated GN. In this case, the diagnosis of IE was confirmed following the modified Duke criteria by isolating this unusual pathogen in three different blood samples, visualizing a vegetation *via* a transesophageal echocardiogram as well as a kidney pathologic diagnosis of diffuse proliferative immune complex-mediated GN.

Despite improvements in diagnosis and treatment, IE carries a high risk of morbidity and mortality, and about 20%–50% of patients require cardiac valve replacement (1, 7). Because the presentation of IE is highly variable and often nonspecific, early recognition and comprehensive management is essential, including a multidisciplinary team approach as well as innovative diagnostic tools with targeted antimicrobial therapy to help prevent poor outcomes (2, 3). Renal involvement can aid in diagnosis of IE. In a large cohort of 49 patients with IE who underwent kidney biopsy, 79% of cases presented with acute kidney injury and 97% had hematuria at presentation (4). Only 21% were diagnosed with acute nephritic, rapidly progressive, or nephrotic syndrome at presentation. It was noted that in this cohort, all subjects underwent kidney biopsy, thus capturing the most serious kidney manifestations with acute kidney injury at presentation. Nonetheless, all suspected cases of IE, even with subtle renal involvement, should be carefully evaluated with a screening urine examination to assess for hematuria and proteinuria. Our patient presented with rapidly progressive GN, which prompted our concern of possible IE due to his underlying valvular heart disease.

The main differential diagnosis of acute glomerulonephritis in this setting includes post-infectious glomerulonephritis (PIGN), which may occur coincidently with *Bordetella holmesii* endocarditis, given his history of preceding acute respiratory tract infection. However, there are several arguments against PIGN. First, typically PIGN occurs following resolution of infection within 1–3 weeks of preceding infection (3). Contrary to this, in our case, glomerulonephritis occurred after 5 weeks of prior respiratory infection and the patient had an ongoing infection. Second, patients with PIGN often have predominant evidence of the activation of alternative pathway from both serum complement profiles and immunofluorescence patterns for glomerular deposits (8), but our case had significant findings of classical complement pathway activation with low circulating complement C3 and C4, a positive C1q staining, along with C3, IgM, and IgG in immunofluorescence. In addition, subepithelial hump, the hallmark electron microscopy for PIGN, was not observed in our case.

Hypocomplementemia is a hallmark finding in IE-associated GN, suggesting complement pathway activation in the pathogenesis; nonetheless, over 40% may have normal complement levels (4). The degree of complement reduction correlates with the severity of renal disease (9, 10). The serum complement level normalizes with appropriate antimicrobial treatment. Therefore, complement levels are an important marker to monitor in these patients. Furthermore, one-third of cases have positive ANCA (3, 4), which might indicate the heterogeneous immunopathogenesis of IE-associated GN due to the broad clinical settings and various pathogens causing IE. Our patient had low complement C3 and C4 levels and a positive ANCA upon presentation, which improved throughout his clinical course with appropriate antibiotic treatment.

Various kidney pathologies have been demonstrated in IE-associated GN, which include crescentic GN, diffuse proliferative GN, focal proliferative GN, and mesangial proliferative GN. Of those who underwent biopsy, crescentic GN was noted to be the most common kidney pathology, followed by diffuse proliferative GN in 33% of cases (3, 4). Our patient's biopsy demonstrated a diffuse proliferative immune complex GN with IgM and C3 codominance, similar to previously reported pathology (3, 4).

Typically, the most common organisms in IE-associated GN are *Staphylococcus* and *Streptococcus* species (1, 3, 4, 7). *Bordetella holmesii* has been shown as an emerging pathogen causing IE with only 11 cases reported (11, 12). Over half of these cases were immunocompromised, mostly due to asplenia or immunosuppressant therapy. Our patient is immunocompetent and has no history of opportunistic infection or evidence of functional asplenia from. None of the cases had IE-associated GN.

*Bordetella holmesii* is a gram-negative, rod-shaped bacillus of the genus *Bordetella*. It is an underrecognized species in *Bordetella* since first isolated in 1983 in a patient with asplenia. It was finally named in 1995 in honor of Barry Homes, a microbiologist who ultimately identified the rare pathogen (13). Since then, it has been reported as a causative pathogen causing bacteremia, meningitis, pericarditis, pneumonia, and arthritis, in addition to pertussis-like symptoms, but mostly in immunocompromised patients (14, 15). Isolation of *Bordetella* species is complicated, and the pathogen is difficult to speciate quickly, as it takes more time to grow, in comparison to more common pathogens. The case also highlights the importance of innovative diagnostic tools, including molecular testing and genomic sequencing in the detection of obscure bacteria causing IE (2, 13–15). In our case, we utilized MALDI-TOF MS and microbial cell-free DNA sequencing to confirm the diagnosis.

The case series describing *B. holmesii* IE discussed that up to 70% of cases had morbid outcomes, including 50% requiring heart valve replacement (11). Our case recognized renal manifestations as the first indicator of possible IE, with a high index of suspicion given the history of prosthetic valve replacement. This early recognition led to a multidisciplinary approach to confirm the diagnosis of IE-associated GN, allowing for preservation of cardiac function and renal recovery.

## Conclusion

We describe a rare pathogen, *Bordetella holmesii*, causing IE which led to the development of immune-mediated GN. A high index of suspicion in a patient at risk of developing IE can allow for an early diagnosis and comprehensive management of IE. Renal manifestations may be the only presentation of IE.

## Data Availability

The original contributions presented in the study are included in the article/Supplementary Material, further inquiries can be directed to the corresponding author.
